# Nav1.9 Channel Contributes to Mechanical and Heat Pain Hypersensitivity Induced by Subacute and Chronic Inflammation

**DOI:** 10.1371/journal.pone.0023083

**Published:** 2011-08-12

**Authors:** Stéphane Lolignier, Muriel Amsalem, François Maingret, Françoise Padilla, Mélanie Gabriac, Eric Chapuy, Alain Eschalier, Patrick Delmas, Jérôme Busserolles

**Affiliations:** 1 Clermont Université, Laboratoire de Pharmacologie Fondamentale et Clinique de la Douleur, Clermont-Ferrand, France; 2 Institut National de la Santé et de la Recherche Médicale, Unité 766, Clermont-Ferrand, France; 3 Université de la Méditerranée, Centre National de la Recherche Scientifique Unité Mixte de Recherche 6231, Centre de Recherche en Neurobiologie et Neurophysiologie de Marseille, Marseille, France; 4 Centre Hospitalier Universitaire de Clermont-Ferrand, Service de Pharmacologie, Clermont-Ferrand, France; Dalhousie University, Canada

## Abstract

Inflammation is known to be responsible for the sensitization of peripheral sensory neurons, leading to spontaneous pain and invalidating pain hypersensitivity. Given its role in regulating neuronal excitability, the voltage-gated Nav1.9 channel is a potential target for the treatment of pathological pain, but its implication in inflammatory pain is yet not fully described. In the present study, we examined the role of the Nav1.9 channel in acute, subacute and chronic inflammatory pain using Nav1.9-*null* mice and Nav1.9 knock-down rats. In mice we found that, although the Nav1.9 channel does not contribute to basal pain thresholds, it plays an important role in heat pain hypersensitivity induced by subacute paw inflammation (intraplantar carrageenan) and chronic ankle inflammation (complete Freund's adjuvant-induced monoarthritis). We showed for the first time that Nav1.9 also contributes to mechanical hypersensitivity in both models, as assessed using von Frey and dynamic weight bearing tests. Consistently, antisense-based Nav1.9 gene silencing in rats reduced carrageenan-induced heat and mechanical pain hypersensitivity. While no changes in Nav1.9 mRNA levels were detected in dorsal root ganglia (DRGs) during subacute and chronic inflammation, a significant increase in Nav1.9 immunoreactivity was observed in ipsilateral DRGs 24 hours following carrageenan injection. This was correlated with an increase in Nav1.9 immunolabeling in nerve fibers surrounding the inflamed area. No change in Nav1.9 current density could be detected in the soma of retrolabeled DRG neurons innervating inflamed tissues, suggesting that newly produced channels may be non-functional at this level and rather contribute to the observed increase in axonal transport. Our results provide evidence that Nav1.9 plays a crucial role in the generation of heat and mechanical pain hypersensitivity, both in subacute and chronic inflammatory pain models, and bring new elements for the understanding of its regulation in those models.

## Introduction

Acute or chronic pathological tissue inflammation strongly impacts on pain perception by sensitizing peripheral sensory neurons, giving rise to local and incapacitating pain hypersensitivity. Inflammatory mediators are known to enhance nociceptive primary afferent fibers excitability, in part by modifying expression and/or function of ionic channels present in nerve endings [1]. Voltage-gated sodium channels (VGSCs) play a fundamental role in neuronal excitability as they are directly responsible for initiation and propagation of action potentials, and their implication in different chronic pain disorders, including inflammatory pain, is relatively well established [2]. Among the 10 VGSC isoforms, two tetrodotoxin-resistant (TTX-R) channels, Nav1.8 and Nav1.9, are almost exclusively expressed in nociceptors, consistently with a specific involvement in nociceptive pathways [3], [4], [5], [6]. Nav1.8 has been shown to generate a slowly-inactivating sodium current with a relatively depolarized activation threshold, underlying the depolarizing phase of action potential in C-type fibers [7], [8], [9]. Several knock-down and knock-out studies have clearly demonstrated its contribution to pain hypersensitivity in both neuropathic and inflammatory models [10], [11], [12], [13], [14]. More recently, a link between Nav1.9 channel and inflammatory pain hypersensitivity has also been established [15], [16].

Nav1.9 channel shows atypical properties. Its voltage-dependent activation is shifted to hyperpolarized potentials compared to Nav1.8 and TTX-sensitive channels [17], and its activation and inactivation curves are widely overlapping around the resting potential, enabling it to produce a persistent sodium current component [18], [19]. Moreover, its slow activation kinetics argues against a contribution of Nav1.9 to the depolarizing phase of the action potential [18], [9]. Therefore, it has been initially proposed that Nav1.9 channel could rather contribute to the setting of the excitability of nociceptive sensory neurons by modulating both resting potential and response to subthreshold stimuli [20]. More recently, it has been shown that inflammatory soup-triggered up-regulation of Nav1.9 current increased the excitability of DRG neurons by generating long-lasting plateau depolarization and burst firing [21]. Other studies performed using different inflammatory protagonists such as prostaglandin E2, protein kinase C or G-proteins, also highlighted the link between inflammatory pathways and Nav1.9-mediated increase in the excitability of nociceptors [22], [23], [24], [25].

This is consistent with the first behavioral observations made on Nav1.9-null mice [15], [16]. Knock-out mice did not show any modification of their normal pain thresholds while both heat and pressure hypersensitivities in response to intraplantar injections of inflammatory mediators (prostaglandin E2, bradykinin or interleukin-1β) were reduced. However, results obtained with inflammatory models remain to be completed and clarified as strong discrepancies exist. Indeed, deletion of Nav1.9 was shown to either diminish [15], [16] or have no effect [26] on thermal pain hypersensitivity produced by carrageenan or complete Freund adjuvant (CFA) injections, and the reduction of mechanical hypersensitivity observed in Nav1.9^−/−^ mice in response to inflammatory mediators was not confirmed in the CFA-induced paw inflammation model [15], [16], [26] that is the only one tested until now. Furthermore, whether Nav1.9 expression is modified during inflammation is also still unclear, as conflicting results have been published in different models, or at different time-points following inflammation, showing either increased [5], [16], [27] or stable [28], [29] Nav1.9 expression.

As to settle those issues, we first performed a time-course study of Nav1.9 involvement in thermal and mechanical pain hypersensitivity, using both subacute and chronic (monoarthritis) inflammatory pain models in Nav1.9 knock-out mice. Mechanical pain was not only assessed by von Frey or paw pressure tests, but also using a newly developed weight bearing test in freely moving animals. To reinforce the robustness of the behavioral results obtained with Nav1.9 knock-out mice, we performed a knock-down study in inflamed rats. Behavioral data were then correlated with molecular and electrophysiological analysis of Nav1.9 regulation in the same inflammatory pain models to allow an overall comprehension of the role played by this channel in pain hypersensitivity along the time-course of inflammation.

We first confirmed that healthy Nav1.9-null mice have no modified heat and pressure pain thresholds. Then we showed that the channel is involved in both heat and mechanical pain hypersensitivity during subacute inflammation, and in the early phase of monoarthritis development. Similar observations were performed in the subacute inflammatory model using knock-down strategy in rats. Behavioral results were not correlated with changes in the channel transcription, but rather with variations of protein levels in DRG cell bodies and with an increase in Nav1.9 transport to the nerve endings surrounding the site of inflammation. No modulation of Nav1.9 current was detected in the soma of neurons innervating the inflamed zone, suggesting that the newly produced channels are non-functional at the cell body level.

## Results

### Reduction of acute inflammatory pain in Nav1.9^−/−^ mice

**Figure 1:**
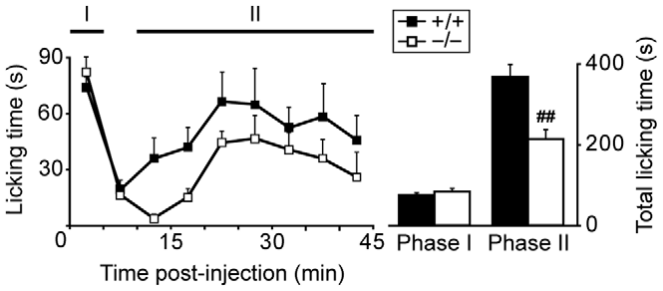
Nav1.9 knock-out mice behavioral response to intraplantar formalin injection. Following intraplantar formalin, the time spent licking/biting the injected paw is monitored during 45 min in Nav1.9^+/+^ (n = 9) and Nav1.9^−/−^ (n = 8) mice. Data were summed for the two typical phases of the test. Phase I: 0 to 5 min. Phase II: 10 to 45 min. ##p<0.01 vs ‘+/+’, Student's unpaired t-test.

Formalin test was performed to assess acute inflammatory pain in this Nav1.9^−/−^ mouse strain (Fig. 1). Following intraplantar formalin injection, mice painful behavior, characterized by the licking or biting of the injected paw, was monitored during the two typical phases of the test (0 to 5 min and 10 to 45 min). During the first and non-inflammatory phase, Nav1.9^−/−^ mice and wild-type (WT) littermates exhibited similar spontaneous pain-related behavior. In contrast, we observed a strong diminution in the paw licking time in Nav1.9 knock-out mice during the second and inflammatory phase of the test, which confirms the role played by Nav1.9 in acute inflammatory pain [15].

### Involvement of Nav1.9 in heat and mechanical pain hypersensitivity induced by subacute inflammation

**Figure 2:**
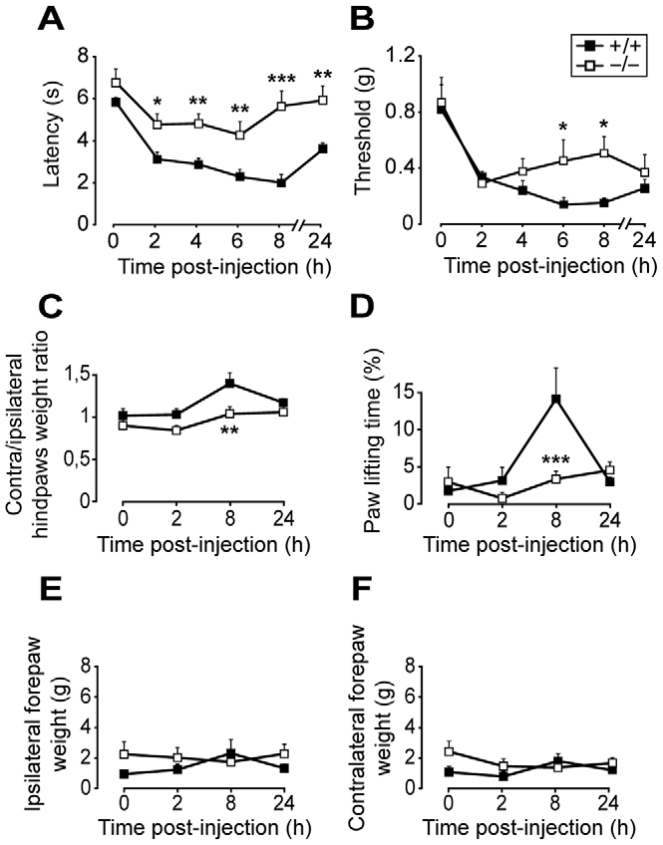
Nav1.9 knock-out mice pain sensitivity and weight imbalance following carrageenan intraplantar injection. Following intraplantar carrageenan, heat and mechanical pain sensitivity of Nav1.9^+/+^ and Nav1.9^−/−^ mice was assessed over 24 h using 46°C paw immersion test (A, n = 9), von Frey test (B, n = 9), and dynamic weight bearing test (C–F, n = 5). The ipsi/contralateral hindpaw weight ratio is shown in C and the ipsilateral paw lifting time in D. The mean weights applied on ipsilateral and contralateral forepaws are also shown in E and F, respectively. *p<0.05, **p<0.01, ***p<0.001 vs ‘+/+’, two way ANOVA followed by Student-Newman-Keuls all pairwise multiple comparison test.

We evaluated the contribution of Nav1.9 channels to mechanical and heat pain hypersensitivity in the carrageenan subacute paw inflammation model. Mice were subjected to different thermal and mechanical pain tests over a 24 h period following intraplantar carrageenan. The intensity of the inflammatory reaction was evaluated in Nav1.9^−/−^ and WT mouse strains by measuring the induced edema. No difference in paw swelling was observed between strains (see supplementary Fig. S1A). Heat pain threshold was evaluated by paw withdrawal latency after immersion in a 46°C water bath (Fig. 2A). Basal responses to noxious heat were identical between the two mouse strains, but following intraplantar carrageenan WT mice developed a strong hypersensitive phenotype (p≤0.003 from 2 to 24 h) that was significantly reduced in Nav1.9^−/−^ mice at all times post-injection.

Similarly, carrageenan injection induced a subacute mechanical hypersensitivity in WT mice (p≤0.003 from 2 to 24 h) which was significantly reduced in Nav1.9^−/−^ mice 6 and 8 h post-injection (Fig. 2B). To confirm the lower hypersensitivity in Nav1.9 knock-out mice, we performed a dynamic evaluation of the weight bearing of animals on their 4 limbs over a 5 min period. Eight hours following carrageenan injection, a slight increase in contra/ipsilateral weight ratio (Fig. 2C), as well as a strong increase in inflamed hindpaw lifting time (Fig. 2D), were observed in WT mice (p = 0.014 and p<0.001, respectively). The weight applied on the forepaws was not affected (Fig. 2E–F). In Nav1.9 knock-out mice, no such weight imbalance or postural alteration was detected (p = 0.247 for contra/ipsilateral weight ratio, p = 0.883 for paw lifting time) and a significant difference between the two strains was also found 8 h post-injection. Taken together, these data demonstrate the involvement of Nav1.9 in mechanical pain hypersensitivity in a subacute model of paw inflammation.

**Figure 3:**
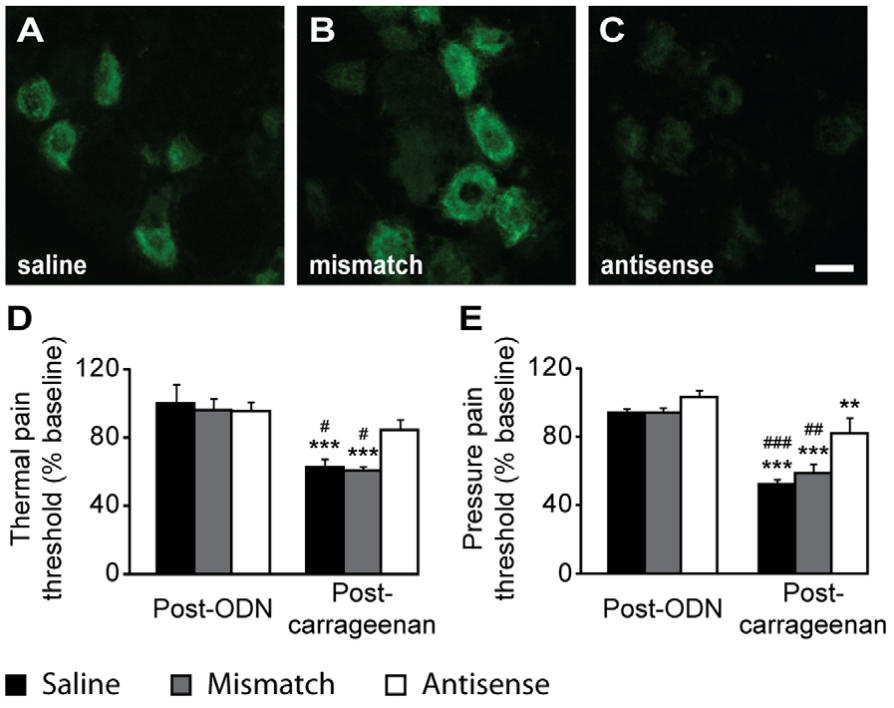
Rats pain sensitivity following Nav1.9 antisense treatment and intraplantar carrageenan injection. Nav1.9 immunolabeling on L5 DRGs from rats treated intrathecally with saline (A), mismatch ODN (B) and antisense ODN (C) for 3 days. Heat (D) and mechanical (E) pain thresholds of rats (n = 8) are evaluated by 46°C paw immersion and paw pressure tests, respectively, before and 4 h after carrageenan intraplantar injection. Pain thresholds are given as percentage from pre-ODN baseline. **p<0.01, ***p<0.001 vs ‘Post-ODN’ ; #p<0.05, ##p<0.01, ###p<0.001 vs ‘Antisense’, two way ANOVA followed by Student-Newman-Keuls all pairwise multiple comparison test. Scale bar 20 µm.

Compensatory mechanisms may occur in knock-out strains, leading to changes in the expression of non-targeted genes, as previously shown in Nav1.8^−/−^ mice in which an increase in TTX-sensitive current density has been observed [11]. To prevent any misinterpretation of the results obtained with Nav1.9^−/−^ mice, we assessed behavioral reactions to noxious stimuli in Nav1.9 knock-down rats following carrageenan paw inflammation. This consisted in intrathecal injections of antisense oligodeoxynucleotides (ODN) complementary to Nav1.9 transcript twice a day during 3 days. Nav1.9 down-regulation by antisense ODN, together with the lack of effect of a mismatch ODN or saline injection, were validated in L5 DRGs by immunohistochemistry (Fig. 3A–C). Following Nav1.9 antisense treatment, no difference was found in heat and mechanical pain thresholds (Fig. 3D–E). Paw inflammation was then induced by intraplantar carrageenan injection. This lowered heat and mechanical pain thresholds of rats, but the observed hypersensitivity was significantly reduced in the Nav1.9-antisense treated group for both tests. These data confirm the involvement of Nav1.9 channel in inflammatory pain hypersensitivity, whatever the sensory modality tested.

**Figure 4:**
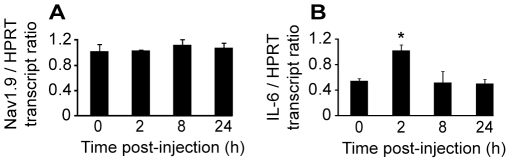
Nav1.9 and IL-6 mRNA expression in mouse lumbar DRGs following intraplantar carrageenan. Quantitative PCR performed on Nav1.9 (A) and IL-6 (B) transcripts in L4 to L6 ipsilateral DRGs of mice following intraplantar carrageenan (n = 3). Results are given normalized to HPRT transcript levels. *p<0.05 vs ‘0’, one-way ANOVA followed by Bonferroni multiple comparisons versus control group.

**Figure 5:**
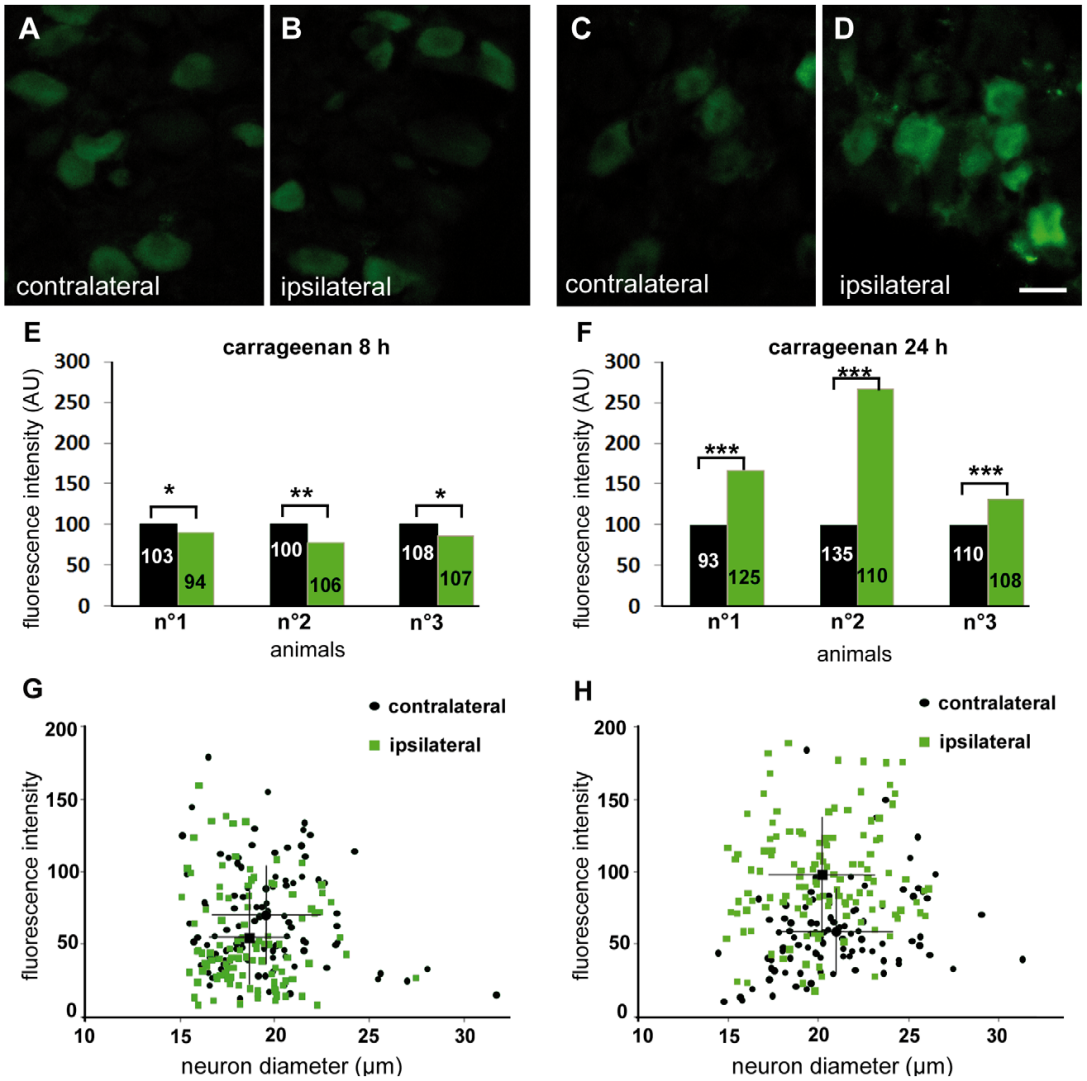
Modulation of Nav1.9 protein level in sensory neuron cell body during carrageenan induced inflammation. Nav1.9 immunolabeling of ipsilateral and contralateral DRG sections from animals, 8 h (A–B) and 24 h (C–D) following injection of 3% carrageenan. Mean fluorescence intensities of ipsilateral and contralateral neurons from 3 different animals are shown in E and F, 8 h and 24 h post-injection, respectively (exact numbers of neurons quantified is indicated in each bar). To better compare fluorescence intensities, mean grey values were normalized setting contralateral neuron populations to 100 AU. Mean grey value and diameter of Nav1.9-labeled neurons were measured in ipsilateral (green squares) and contralateral (black circles) DRGs and plotted into graphs (G–H). Large black square and circle in each graph represent the mean ± SEM intensity/diameter coordinates of ipsilateral and contralateral neurons populations, respectively. Scale bar 20 µm. *p<0.05, **p<0.01, ***p<0.001 vs ‘contralateral’, Mann and Whitney rank test.

**Figure 6:**
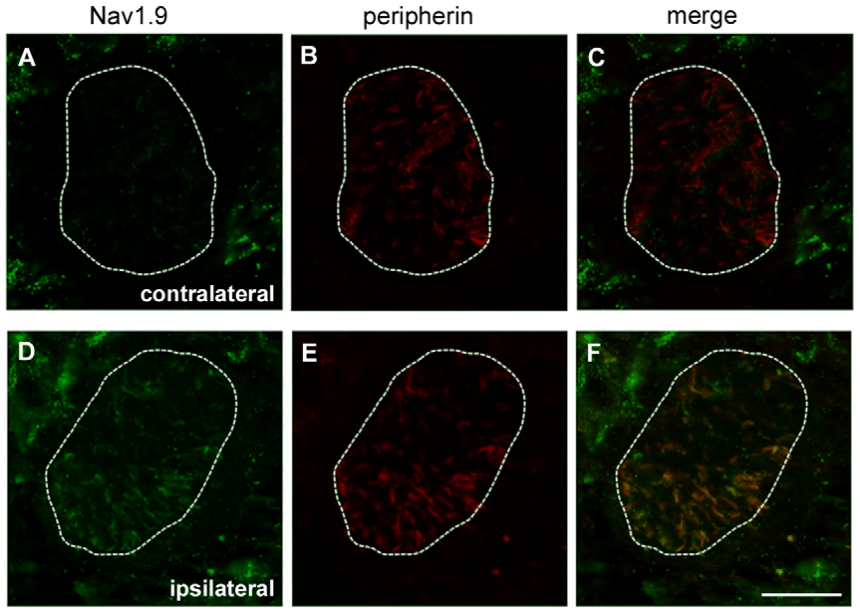
Modulation of Nav1.9 protein level in cutaneous sensory fibers innervating the carrageenan-inflamed paw. Co-labeling of Nav1.9 (A, D) and peripherin (B, E) in small cutaneous nerve trunks in contralateral (A–C) and ipsilateral (D–F) paw skin 24 h after intraplantar carrageenan injection. Merge images are shown in C and F. Scale bar 28 µm.

We then performed a time course study of Nav1.9 transcript and protein expression in L4 to L6 DRGs by quantitative RT-PCR and immunohistochemistry, respectively. No variation in Nav1.9 transcript level was found at any time post-carrageenan (Fig. 4A). IL-6 transcript was assessed as a positive control, and a two fold increase was detected in ipsilateral DRGs 2 h following carrageenan injection (Fig. 4B). A slight decrease (around 15%) of Nav1.9 immunolabeling was observed 8 h after carrageenan injection in ipsilateral neurons compared to contralateral ones (Fig. 5A–B,E). Such a decrease is significant in every tested animal and was not observed in saline injected animals at the same time point (data not shown). 24 h after injection, Nav1.9 immunolabeling was increased in ipsilateral DRG neurons compared to contralateral ones (Fig. 5C–D,F). Increase in Nav1.9 immunolabeling was already detectable 16 h post-injection (data not shown). We then assessed the size of Nav1.9-positive neurons since some pathological processes have already been reported to induce Nav1.9 expression in larger DRG neurons [30]. The distribution of Nav1.9 labeling intensity plotted against the size of neurons did not show any difference at any time point tested between contra and ipsilateral DRG, as illustrated in Fig. 5G–H. To test whether the transient decrease in Nav1.9 protein level in DRG neuron somata resulted in an increase in Nav1.9 transport to peripheral nerve endings, we searched for an accumulation of Nav1.9 protein in inflamed territories. As it is nearly impossible to have an exhaustive quantification of Nav1.9 protein level in sensory endings within the epidermis, we rather looked at small nerve trunks in the vicinity of the epidermis. Such trunks correspond to very distal part of the sensory fibers and were identified by the peripherin marker (Fig. 6B,E). Twenty-four hours after carrageenan injection, a marked accumulation of Nav1.9 protein was observed in nerve trunks surrounding the inflamed skin (Fig. 6).

**Figure 7:**
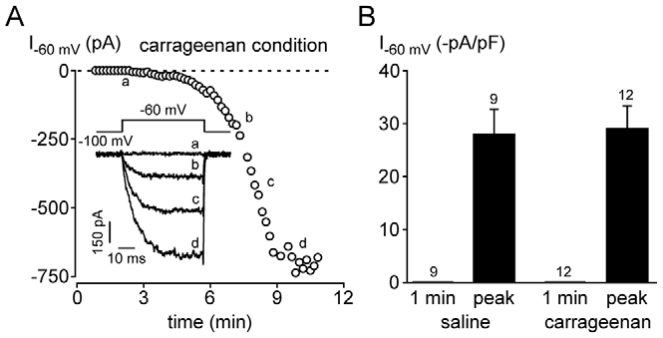
Nav1.9 current properties in the carrageenan-induced paw inflammation model. (A) Time-course of Nav1.9 current up-regulation induced by dialysis of fluoride in the carrageenan condition. Nav1.9 currents are evoked in a DRG neuron (25 pF) by 50 ms-depolarizing steps from −100 to −60 mV every 10 s. The inset shows representative Nav1.9 currents recorded during the time course of the experiment (as indicated). (B) Mean Nav1.9 current evoked at −60 mV 1 min after patch rupturing and at the peak of the up-regulation by internal fluoride. The experiments were performed in DRG neurons from mice injected either with saline (control condition, left panel) or carrageenan (inflammatory condition, right panel). The number of cells analyzed is shown on the top of each chart.

Finally, we investigated whether Nav1.9 current was potentiated in the carrageenan subacute inflammation model. To this aim, 24 h following intraplantar carrageenan injection we performed whole-cell patch clamp recordings from cultured small DRG neurons (24±2 pF, n = 21) that were innervating the inflamed zone, as seen by retrograde labeling with the fluorescent dye DiI that was previously injected intraplantarly. Nav1.9 current is typically recorded at a test potential of −60 mV using fluoride in the pipette solution [18], [19]. Under these experimental conditions, little if any Nav1.9 channel activity could be detectable immediately after achieving the whole cell configuration. The Nav1.9 current developed as fluoride diffuses into the cell. In carrageenan condition, no Nav1.9 current was detectable at −60 mV, 1 min after the rupture of the membrane. However, as fluoride diffuses, Nav1.9 currents could be recorded within minutes (time to peak: 9.8±1.2 min, n = 12, Fig. 7A). This up-regulation of Nav1.9 by fluoride was thereafter compared to the control condition (*i.e.* saline injected-mice). As in the carrageenan condition, Nav1.9 current was first not detectable at −60 mV, 1 min after achieving the whole cell configuration, but was up-regulated by internal fluoride within minutes (time to peak: 8.9±1.0 min, n = 9, Fig. 7B). Neither the current amplitude measured at −60 mV (−28.1±4.8 pA/pF, n = 9 vs −29.2±4.3 pA/pF, n = 12) nor the current activation threshold (−71.7±1.5 mV, n = 9 vs −74.2±0.8 mV, n = 12) were significantly different between saline and carrageenan conditions, respectively, suggesting that the observed increase in Nav1.9 expression in cell bodies 24 h following carrageenan injection involves non-functional channels.

### Involvement of Nav1.9 in chronic inflammatory pain in monoarthritic mice

**Figure 8:**
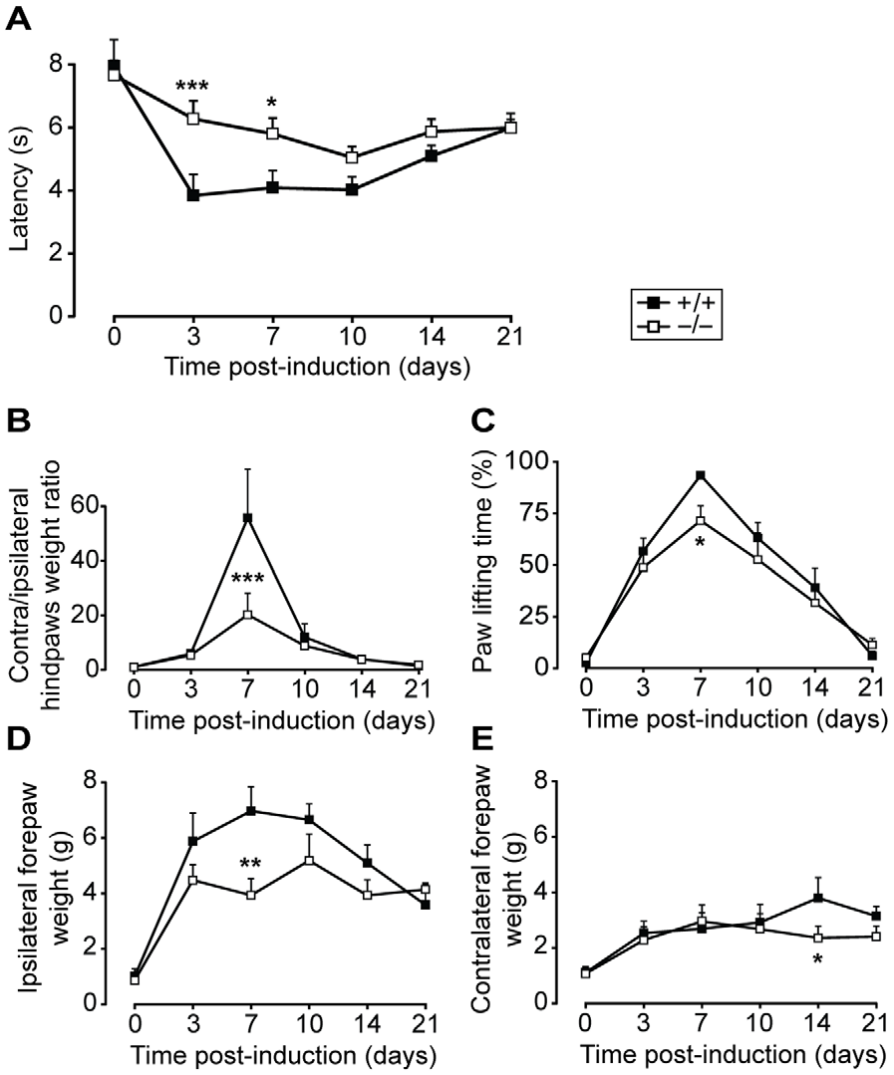
Pain assessment in Nav1.9 knock-out mice following monoarthritis induction. Heat and mechanical pain sensitivity was assessed in Nav1.9^+/+^ and Nav1.9^−/−^ mice (n = 8) over 21 days following monoarthritis induction using 46°C paw immersion test up to the ankle (A) and dynamic weight bearing (B–E). Animals were injected in the left ankle. The ipsi/contralateral hindpaw weight ratio is shown in C and the ipsilateral paw lifting time in D. The mean weights applied on ipsilateral and contralateral forepaws are also shown in E and F, respectively. *p<0.05, **p<0.01, ***p<0.001 vs ‘+/+’, two way ANOVA followed by Student-Newman-Keuls all pairwise multiple comparison test.

To assess whether Nav1.9 channel also contributes to chronic inflammatory pain, we performed a time-course study of Nav1.9^−/−^ and WT mice pain thresholds following monoarthritis induction by two peri-articular CFA injections on either side of the left ankle. Ankle swelling did not differ between the two mouse strains (see supplementary Fig. S1B). As for the intraplantar carrageenan model, heat pain threshold was evaluated by paw immersion test in a 46°C water bath, up to the ankle. This model resulted in a strong hypersensitivity to heat, characterized by the reduction of paw withdrawal latencies from day 3 to day 21 in WT animals (p≤0.007, Fig. 8A). A significant reduction in heat hypersensitivity was observed in Nav1.9^−/−^ mice compared to the WT strain from day 3 to day 7, similarly to what we obtained in WT mice treated by daily intraperitoneal indomethacin injections (data not shown). Mechanical pain, traduced by ankle incapacitance, was also evaluated using the dynamic weight bearing. In WT mice, we observed a strong increase in contra/ipsilateral weight ratio (p<0.001 at day 7 only, Fig. 8B) as well as in inflamed hindpaw lifting time (p<0.001 from day 3 to 14, non-significant at day 21, Fig. 8C), with an acme 7 days following monoarthritis induction. Anteroposterior weight balance was also affected as a strong increase in ipsilateral (p≤0.007 from day 3 to 21, Fig. 8D) and to a less extent contralateral (p≤0.050 at days 3, 10, 14 and 21, Fig. 8E) forepaws weight could be detected, with a peak at day 7 and day 14, respectively. All these observations were also made on Nav1.9 knock-out mice, but a significant reduction in the phenotype intensity was observed at day 7 for the ipsi/contralateral weight ratio, the inflamed paw lifting time and the ipsilateral forepaw weight, and at day 14 concerning the contralateral forepaw. Taken together, these data show that Nav1.9 channels contribute to heat as well as mechanical arthritic pain.

**Figure 9:**
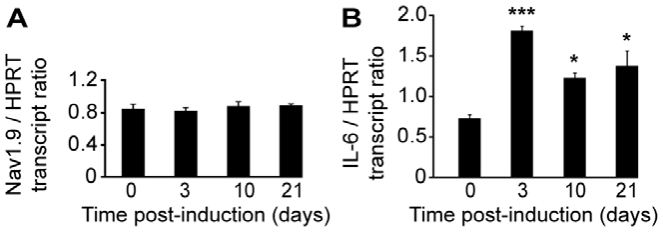
Nav1.9 and IL-6 mRNA expression in mouse lumbar DRGs following monoarthritis induction. Quantitative PCR performed on Nav1.9 (A) and IL-6 (B) transcripts in L4 to L6 ipsilateral DRGs of mice following monoarthritis induction (n = 3). Results are given normalized to HPRT transcript levels. *p<0.05, ***p<0.001 vs ‘0’, one-way ANOVA followed by Bonferroni multiple comparisons versus control group.

In this model, quantitative RT-PCR on L4 to L6 DRGs did not show any variation in Nav1.9 transcript level from day 0 to day 21 (Fig. 9A) whereas IL-6 transcript, used as a positive control, was significantly increased from day 3 to 21 (Fig. 9B). Seven and ten days following monoarthritis induction, no significant change in Nav1.9 immunolabeling was detected in ipsilateral DRG neurons compared to contralateral neurons (data not shown).

**Figure 10:**
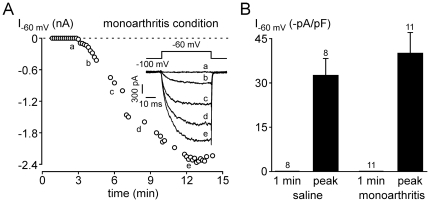
Nav1.9 current properties in the monoarthritis ankle inflammation model. (A) Time-course of Nav1.9 current up-regulation induced by dialysis of fluoride in the monoarthritis condition. Nav1.9 currents are evoked in a DRG neuron (38 pF) by 50 ms-depolarizing steps from −100 to −60 mV every 10 s. The inset shows representative Nav1.9 currents recorded during the time course of the experiment (as indicated). (B) Mean Nav1.9 current evoked at −60 mV 1 min after patch rupturing and at the peak of the up-regulation by internal fluoride. The experiments were performed in DRG neurons from control (injected with saline, left panel) or monoarthritic mice (right panel). The number of cells analyzed is shown on the top of each chart.

We finally investigated whether Nav1.9 channel activity was modulated in the monoarthritic chronic inflammation model. We performed the same experiments as with the carrageenan model, *i.e.* whole-cell patch clamp recordings from cultured small DRG neurons (23±2 pF, n = 19) removed from animals 7 days following monoarthritis induction. Only cells stained by retrograde labeling with the fluorescent dye DiI (injected around the tibio-tarsal joint) were considered. In monoarthritis condition, no Nav1.9 current is detectable, at −60 mV, 1 min after the rupture of the membrane. After several minutes (time to peak: 11.6±0.8 min, n = 11), Nav1.9 current was up-regulated by internal fluoride (Fig. 10A) with a mean current amplitude measured at −60 mV of −40.1±6.9 pA/pF (n = 11) and a current activation threshold of −75.4±0.8 mV (n = 11). In control condition, Nav1.9 current was also undetectable, at −60 mV, 1 min after achieving the whole cell configuration (Fig. 10B) and then reached a maximum amplitude at −60 mV of −32.5±5.7 pA/pF (n = 8) within 12.2±0.7 min (n = 8). Nav1.9 activation threshold was −73.7±0.8 mV (n = 8) in control condition, which was undistinguishable from the activation threshold measured in the monoarthritis condition.

## Discussion

In the present study, we investigated whether Nav1.9 channel contributes to acute, subacute and chronic inflammatory pain in mice and rats using knock-out and knock-down strategies, respectively. We showed that whereas Nav1.9 is not involved in basal pain thresholds, it plays an important role in acute inflammatory pain, as well as in both mechanical and heat pain hypersensitivities produced by subacute paw and chronic joint inflammation. In the subacute inflammatory pain model, these behavioral observations were correlated with post-transcriptional modulations of Nav1.9 expression in DRG cell bodies, and with a strong upregulation of the protein in the peripheral nerve trunks. Furthermore, at a time where Nav1.9 was strongly overexpressed in DRG cell bodies, no Nav1.9 current increase could be detected, suggesting that the newly produced channels are non-functional and rather intended to be exported to nerve terminals.

The relationship between Nav1.9 and inflammatory pain has previously been pointed out by different studies, but this is the first time that one correlates behavioral, molecular and electrophysiological data on the same inflammatory models, with time-course monitoring. These previous studies also brought out some discrepancies that needed to be clarified. Firstly, heat hypersensitivity induced by intraplantar carrageenan [15], [26] or CFA [15], [16], [26] have been reported to be either reduced [15], [16] or unaffected [26] in Nav1.9-null mice. Our results show that Nav1.9 is involved in the decrease in inflammatory heat pain threshold following carrageenan injection. In the chronic monoarthritis model, Nav1.9 invalidation also weakened heat hypersensitivity phenotype, but mostly during the first week of monitoring. Those results confirm the role of Nav1.9 channel in establishing inflammatory heat pain hypersensitivity, and to a lower extent, in its maintenance.

On the other hand, it has previously been shown that Nav1.9 was not involved in inflammatory mechanical hypersensitivity following intraplantar CFA [15], [16], [26]. However, this has never been assessed using a different inflammatory pain model, with the exception of colorectal distention following colonic TLR7 activation [31]. In this last study, Nav1.9 knock-out mice presented a reduction of visceral hypersensitivity to mechanical stimulation. Furthermore, a reduction of mechanical pain hypersensitivity induced by intraplantar injections of inflammatory mediators (prostaglandin E2, bradykinin or interleukin-1β) has been observed in Nav1.9^−/−^ mice [16]. The role played by Nav1.9 in mechanical hypersensitivity driven by inflammation is therefore far from clear and further exploration was needed. Following intraplantar carrageenan injection, our Nav1.9^−/−^ mice exhibited reduced mechanical hypersensitivity compared to WT littermates. This phenotype, observed with the von Frey test also used in the previously cited studies [15], [16], [26], was confirmed with the use of a new operator-independent dynamic weight bearing test. Moreover, our data are supported by the reduction of carrageenan-induced mechanical hypersensitivity observed in Nav1.9 knock-down rats. Finally, we observed a decrease in weight imbalance in monoarthritic knock-out mice, showing that Nav1.9 invalidation is also able to reduce chronic joint pain and the resultant posture impairment. The negative results previously published on mechanical hypersensitivity [15], [16], [26] may be inherent to the intraplantar CFA model. Such discrepancies between inflammatory models have already been reported between carrageenan and CFA for example, considering either the efficiency of pharmacological P2X_3_/P2X_2/3_ receptors inhibition [32], or ASIC3 gene deletion [29], on pain hypersensitivity. Although we could not generalize our conclusions to all the existing inflammatory pain models, taken together our behavioral data give numerous and strong evidences that Nav1.9 participates in lowering heat as well as mechanical pain thresholds during inflammatory states.

In the second part of this study, we assessed Nav1.9 expression in both carrageenan and monoarthritis pain models, as many different studies have reported increased, decreased or stable Nav1.9 mRNA and/or protein expression following peripheral inflammation [5], [28], [33], [16], [27], [29]. Those fragmented information were furthermore rarely correlated with behavioral data, which is an issue for the functional interpretation of possible expression variations. We therefore performed a time-course study of Nav1.9 expression allowing a correlation with the phenotype observed in animal.

A transient decrease in Nav1.9 protein level was observed in the soma of DRG neurons 8 h following intraplantar carrageenan, whereas upregulation was seen 16–24 h post-injection. These changes in somatal protein level were paralleled with an increase in cutaneous nerve trunks within the inflamed zone 24 h following carrageenan injection. These observations strongly suggest that upon inflammation Nav1.9 protein is exported to the periphery, depleting the cytoplasmic pool of channels present in the cell body of sensory neurons, before an increase in its neosynthesis. No such variation was observed in the monoarthritis model, suggesting either that the observed mechanism takes place during the subacute phase of inflammation only, or that a balance between Nav1.9 production and axonal transport has been reached within the chronic phase. As no change in Nav1.9 current properties was detected in DRG neurons innervating the inflamed paw at a time where Nav1.9 was shown to be upregulated, one can assume that a non-functional pool of channels is kept at the soma level and contributes to the increase in their axonal transport. It has been previously demonstrated that when an inflammatory soup is applied on cultured sensory neurons, the cells present a hyperexcitable behavior involving a strong upregulation of Nav1.9 current [21]. Therefore, we hypothesize that the increase in peripherally located channels may contribute to fibers' excitability directly at the site of inflammation.

Nav1.9 modulation by inflammation could temporally be divided in two steps. First, we propose that a rapid modulation of Nav1.9 channels takes place in axon terminals. This idea is supported by the fact that Nav1.9 is present in nerve endings in physiological conditions [34] and by the fact that in the formalin test Nav1.9 is involved in spontaneous painful behavior as soon as 10 to 15 min post-injection. Nav1.9 was also shown to be involved in mechanical hypersensitivity induced by intraplantar PGE_2_ within 15 min [16], and previous electrophysiological studies have revealed rapid modulation of Nav1.9 current following GPCR activation, possibly via a PKC dependent pathway [22], [23], [24], [25]. Secondly, an increase in axonal transport, followed by an upregulation of Nav1.9 channel expression in cell bodies, might take place within the first hours of inflammation as to sustain the excitability of nociceptive terminals.

Changes in Nav1.9 protein expression were not subsequent to modulations in mRNA level, as Nav1.9 transcript remained stable at all time-points tested. Post-transcriptional regulation of VGSCs has been reported previously. For example, Nav1.8 protein but not the corresponding mRNA has been shown to be upregulated in DRG neurons following colonic inflammation [35]. The non-selective cation channel TRPV1 has also been shown to be massively and unilaterally exported to the periphery 24 h following intraplantar CFA [36], sustained by a post-transcriptional upregulation of the channel expression in DRG cell bodies. This type of regulation may occur at different levels, such as dysregulation of mRNA stability, as shown for the Nav1.7 channel [37], or modulation of its translation by RNA binding proteins, as for the ENaC channel [38].

Regarding to these results, Nav1.9 channel may potentially be a suitable pharmacological target for inflammatory pain care. Complete loss of function, as well as partial silencing, were both able to reduce inflammatory pain hypersensitivity, not depending on the nature of the noxious stimuli, while those treatments had no effect on pain thresholds in healthy animal. Thus, drugs inhibiting Nav1.9 should be considered as potential anti-hyperalgesics rather than analgesics, which is conceptually innovative.

## Materials and Methods

### Animals

All animals were used in accordance with the European Community guiding in the care and use of animals (86/609/CEE). Since pain might result from these experiments, the guidelines of the Committee for Research and Ethical Issue of the International Association for the Study of Pain [39] were followed. Furthermore, the models used in this study were all approved by the ethics committee of Auvergne (Comité Régional d'Ethique en Matière d'Expérimentation Animale Auvergne; approvals CE13-10, CE09-08 and CE07-08). Great care was taken, particularly to housing conditions, to avoid or minimize discomfort of the animals. Animals were housed under controlled environmental conditions and kept under a 12/12 h light/dark cycle, with food and water *ad libitum*.

Nav1.9 knock-out mice were generated from C57Bl6/J strain as previously described [21]. Briefly, an IRES LacZ-pA cassette starting by a stop codon was inserted between exons 5 and 8 of the *Scn11a* gene, resulting in a depletion of exons 6 and 7 and the production of a non-functional, truncated protein. Behavioral experiments were performed blind to genotype on Nav1.9^+/+^ and Nav1.9^−/−^ littermates (8–14 weeks) isolated after genotyping on hair samples. Non behavioral experiments were performed on 20–24 g C57Bl6/J mice (Charles River).

Antisense studies were performed on 150–175 g Sprague Dawley rats (Charles River). Prior to experiments, animals received 6 intrathecal injections every 12 h of saline, 12.5 µg mismatch (5′-GCCTTGTCTTTGGACTTCTTC-3′) or antisense oligodeoxynucleotides to Nav1.9 (5′-GCTCTGTTCTTGAGCTTTCTC-3′) in 10 µl saline [12].

### Inflammatory models

Subacute paw inflammation was induced by intraplantar injection of 20 and 50 µl of 3% λ-carrageenan (Sigma) in mice and rats, respectively. Chronic inflammation (monoarthritis model) was induced under 2% isoflurane anesthesia by two subcutaneous injections of 15 µl around the tibio-tarsal joint of mice with complete Freund adjuvant (CFA), containing 5 µg/µl heat-killed *Mycobacterium tuberculosis* (Becton, Dickinson and cie) [40]. Indomethacin (Sigma) was injected intraperitonealy at a 1 mg/kg dose from a 0.3 mg/ml suspension in 0.5% methylcellulose.

### Behavioral assays

#### Formalin test

Mice were administered 15 µl of 5% formalin in saline subcutaneously in the left hindpaw. The time spent liking or biting the injected paw was monitored during the two typical phases of nociceptive behavior (0–5 min and 10–45 min post-injection).

#### Von Frey test

Mechanical pain threshold was assessed using von Frey filaments (BioSeb) calibrated from 0.008 to 4.000 g. The filaments were applied 5 times each, in order of increasing forces, and pressed perpendicularly to the plantar surface of the hindpaw until they bent. The first filament which evoked at least 3 responses was assigned as the pain threshold in grams.

#### Dynamic weight bearing

The animals' weight distribution on the four limbs was assessed using the dynamic weight bearing test (BioSeb). This new incapacitance test consists in a continuous measurement of all pressure points applied by a freely moving animal, allowing a quantitative evaluation of the weight imbalance caused by paw mechanical allodynia. Mice were placed in an 11×11×22 cm cage with a 44×44 sensor cells grid on the floor. The pressure applied on sensor cells by the animal's paws is recorded at a 10 Hz frequency over a 5 min period. Pressure and surface detection thresholds were determined automatically for each animal by the Dynamic weight bearing 1.3.2h software (Bioseb). After the manual attribution of each pressure point to the corresponding paw, the mean weight applied on each paw is calculated. Unilateral pain is finally evaluated trough the ipsi/contralateral hindpaws weight ratio, the weight applied on forepaws, and the percentage of time spent raising ipsilateral hindpaw.

#### Paw pressure test

The rats were submitted to the paw-pressure test previously described by Randall and Selitto [41]. Nociceptive thresholds, expressed in grams, were measured using an Ugo Basil analgesimeter (probe tip diameter 1 mm, Bioseb) by applying an increasing pressure to the hindpaw until vocalisation was elicited. The maximal pressure was set at 450 g.

### Paw immersion test

To assess heat pain threshold, mice and rats were gently held and the hindpaw was immersed in a 46°C water bath until withdrawal was observed. The first two consecutive stable withdrawal latencies were averaged out and assigned as the pain threshold in seconds. A cutoff time of 15 s was applied to avoid injury.

### Nav1.9 expression analysis

#### Real-time PCR

Mice were sacrificed by decapitation under isoflurane anesthesia. Ipsilateral and contralateral DRGs from lumbar segments L4 to L6 were rapidly dissected in 4°C phosphate buffer saline (PBS) and stored at −80°C for subsequent RNA extraction. To ensure sufficient RNA quantity, each assessed sample is the result of a 5 animal DRGs pool. RNA was isolated using RNeasy mini kit (Qiagen) according to the manufacturer instructions, including a 15 min DNase incubation step. Purified RNA was quantified by measuring the 260 nm absorbance (A260) on a V-550 spectrophotometer (Jasco) and quality was assessed by analyzing the A260/A280 and A260/A230 ratios. Integrity of RNA samples was confirmed by electrophoresis on a 1.5% agarose gel. Before quantitative PCR analysis, 1 µg of total RNA was submitted to reverse transcription with SuperScript II Reverse Transcriptase (Invitrogen) in a 20 µl volume, using the supplier's procedure. PCR amplifications were performed using a Mastercycler ep realplex (Eppendorf). All samples were run in triplicate in a final volume of 6.3 µl containing 1.3 µl of 1/40 diluted cDNA, 0.5 µM primers, 3 mM MgCl_2_ and LightCycler Fast-Start SYBR Green reaction mix (Roche), according to manufacturer protocol. Prior to PCR, an 8 min enzyme activation step was done at 95°C. The PCR protocol consisted of 10 s denaturation at 95°C, 5 s at annealing temperature and 10 s elongation at 72°C for 40 cycles. Optimal annealing temperature was preliminary determined by gradient PCR. The primers sequences used were the following, from 5′ to 3′: TTCCACTCTACGTACCTTCCGAGT (forward) and ATTCCCATGAAGAGCTGCTGACCA (reverse) for Nav1.9 (NM_011887, 180 bp); ATGGATGCTACCAAACTGGAT (forward) and TGAAGGACTCTGGCTTTGTCT (reverse) for IL-6 (NM_031168, 139 bp); TTGCTGACCTGCTGGATTAC (forward) and AGTTGAGAGATCATCTCCAC (reverse) for HPRT (NM_013556, 149 bp). Amplification specificity was assessed by melting curve analysis and PCR products were run on a 3% agarose gel to confirm amplicons sizes. Primers were designed on intron-flanking sequences to prevent genomic contamination. Nav1.9 and HPRT cDNA relative amounts were calculated function of the samples cycle threshold (Ct) using a standard concentration curve constructed with a serial dilution from 1/10 to 1/640 of a total cDNA mix. For each sample, the relative amount of Nav1.9 cDNA was normalized by the HPRT cDNA amount.

#### Immunohistochemistry

Beforehand dissection, animals were assessed for mechanical hypersensitivity using von Frey test as described above. DRG and skin samples were quickly removed from freshly killed animals and incubated for 1 h in PBS containing 4% sucrose, and then incubated for at least one hour in PBS plus 15% sucrose. Tissues were then embedded in Tissue-TEK OCT compound (Miles Inc.) and frozen in −60°C dry-ice cooled isopentane. 14 µm thick serial sections were performed with a HM500M Microm cryostat and set down on Superfrost Plus slides. Unspecific binding was reduced by pre-incubating samples in blocking buffer consisting of 3% Bovine Serum Albumin (BSA) and 0.1% triton X-100 in PBS for 1 h. Primary antibodies were incubated in blocking buffer without triton X-100 overnight at 4°C. After several washes in PBS, secondary antibodies were incubated in PBS containing 3% BSA for 1 h at room temperature. After several washed in PBS, sections were mounted in Mowiol. Antibody dilutions were: anti-Nav1.9 L23, 1/400 [34] ; anti-peripherin, 1/400 (Chemicon). Goat anti-rabbit IgG Alexa Fluor 488 (1/400, Molecular Probes) and goat anti-mouse IgG Alexa Fluor 546 (1/400, Molecular Probes) were used as secondary antibodies. Optical conventional fluorescence microscopy was performed on an Olympus microscope equipped with CellR acquisition software (Olympus). Structured illumination microscopy, for skin images, was performed on the ApoTome system (Zeiss). Image editing was performed using Adobe Photoshop (Adobe Systems).

#### Flurorescence quantification

Nav1.9 fluorescence signal was quantified with cellR software (Olympus). For each animal, immunostaining of ispi and contralateral DRG sections were performed simultaneously with the same batch of antibody. Pictures were recorded with the same acquisition parameters, paying attention to avoid any saturation of the signal. Pictures were taken as 8 bit images with 256 (from 0 to 255) possible grey levels. Around a hundred neurons were analysed for each contra and ipsilateral DRG. Mean grey value was measured on a 5.3 µm diameter ROI (Region Of Interest). For each image, mean background was also measured with three ROI, and subtracted to the Nav1.9 fluorescence signal.

### Electrophysiology

#### Acutely dissociated DRG cells

DRG neurons innervating skin from young male mice were labeled by the fluorescent dye DiI (5 repetitive subcutaneous injections of 1 µl in 5% DMSO, Molecular Probes). Dye was injected 3 days before injection of saline, carrageenan, or CFA, depending of the considered model. Beforehand dissection, mechanical hypersensitivity was assessed as described above. In the carrageenan and monoarthritis models, mice were sacrificed respectively 20 h and 7 days after the induction of the inflammation. Dissociation of DRG neurons was performed as been previously described [42], [21]. Briefly, lumbar ipsilateral L4 to L6 DRGs were dissected and incubated in Hank's balanced salt solution (HBSS, Invitrogen) containing collagenase IA (2 mg/ml, Sigma) for 45 min at 37°C. DRGs were then triturated using a fired polished Pasteur pipette in HBSS medium and cultured in Dulbecco's modified Eagle's medium (DMEM, Invitrogen) supplemented with heat-inactivated fetal calf serum (10%), penicillin-streptomycin (100 units/ml), L-glutamine (2 mM), Nerve Growth Factor (25 ng/ml, Gibco) and Glial Derived Neurotrophic Factor (2 ng/ml, Gibco). Cells were maintained in a humidified atmosphere (5% CO_2_, 37°C) for 3–6 h before recording.

#### Patch clamp recordings

Whole-cell voltage clamp recordings were made at room temperature (20–24°C) using an Axopatch 200B amplifier (Axon Instruments), filtered at 2 kHz and digitally sampled at 20–50 kHz using PCLAMP 8.02 software. Currents were leak subtracted using a P/6 protocol. Voltage errors were minimized using 70–85% series resistance compensation. Patch pipettes (1.9–2.5 MΩ) contained (in mM): 100 CsCl, 30 CsF, 8 NaCl, 1 CaCl_2_, 1 MgCl_2_, 10 EGTA, 10 Hepes, 4 MgATP, 0.4 Na_2_GTP (pH 7.2, 300 mOsm/l). Bath solution had a reduced driving force for Na^+^ and contained (in mM): 60 NaCl, 110 sucrose, 3 KCl, 1 MgCl_2_, 10 Hepes, 2.5 CaCl_2_, 10 glucose, 10 TEA-Cl, 0.0005 TTX, 0.005 LaCl_3_, 1 amiloride (pH 7.4, 305 mOsm/l). All chemicals were obtained from Sigma except TTX (Alomone Labs). DRG neurons were perfused with bath solution at a flow rate of 2.5 ml/min.

### Data analysis

Raw data were analyzed for statistical significance using either one-way ANOVA followed by Bonferroni multiple comparisons versus control group, two-way ANOVA followed by Student-Newman-Keuls all pairwise multiple comparison procedure, Student's unpaired t-test or Mann-Whitney test (SigmaPlot) depending on the experimental design. For a better understanding, the tests used are indicated in the corresponding figure legend. All values are shown as mean ± standard error of the mean (SEM).

## Supporting Information

Figure S1 **Carrageenan and monoarthritis-induced oedema monitoring in Nav1.9^−/−^ mice.** Paw edema induced by intraplantar carrageenan was measured from 0 to 24 h post-injection using a micrometer in Nav1.9^−/−^ and Nav1.9^+/+^ mice (A, n = 9). Ankle swelling following monoarthritis induction was measured from 0 to 21 days post-CFA injection in Nav1.9^−/−^ and Nav1.9^+/+^ mice (B, n = 8). No significant difference occurred between genotypes in both models, assessed by two-way ANOVA followed by Student-Newman-Keuls all pairwise multiple comparison test.
(TIF)
