# Supplementary figures and images for: Nav1.9 Channel Contributes to Mechanical and Heat Pain Hypersensitivity Induced by Subacute and Chronic Inflammation
Source: PLoS One. 2011 Aug 12;6(8):e23083. doi: 10.1371/journal.pone.0023083 (PMC3155549; doi:10.1371/journal.pone.0023083)

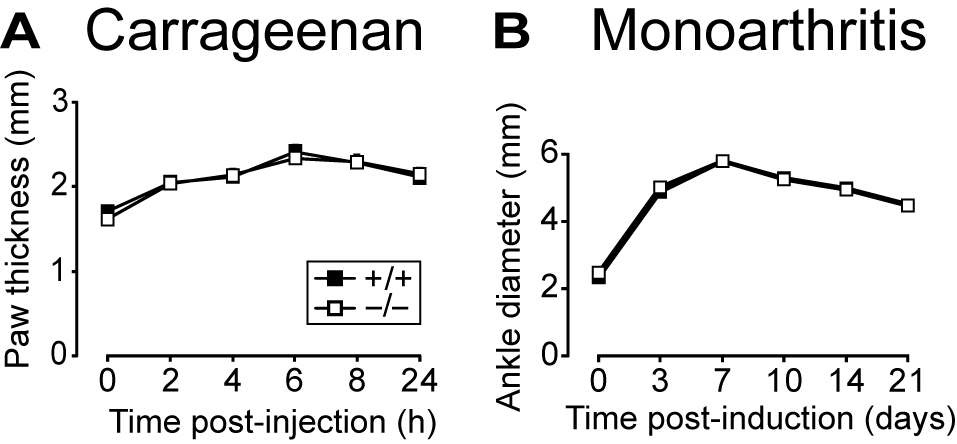

Supplement: Figure S1 — Carrageenan and monoarthritis-induced oedema monitoring in Nav1.9−/− mice. Paw edema induced by intraplantar carrageenan was measured from 0 to 24 h post-injection using a micrometer in Nav1.9−/− and Nav1.9+/+ mice (A, n = 9). Ankle swelling following monoarthritis induction was measured from 0 to 21 days post-CFA injection in Nav1.9−/− and Nav1.9+/+ mice (B, n = 8). No significant difference occurred between genotypes in both models, assessed by two-way ANOVA followed by Student-Newman-Keuls all pairwise multiple comparison test. (TIF) [file pone.0023083.s001.tif]
